# Non-destructive environmental DNA extracted from owl pellet contents: A valuable tool for monitoring mammalian species richness

**DOI:** 10.1371/journal.pone.0344097

**Published:** 2026-03-09

**Authors:** Pia Schoenefuss, Paul Whatmore, Craig Windell, Andrew M. Baker, David A. Hurwood

**Affiliations:** 1 School of Biology and Environmental Science, Queensland University of Technology, Brisbane, Queensland, Australia; 2 Bush Heritage Australia, Melbourne, Australia; 3 eResearch, Research Infrastructure, Academic Division, Queensland University of Technology, Brisbane, Queensland, Australia; 4 Biodiversity and Geosciences Program, Queensland Museum, South Brisbane, Queensland, Australia; UTRGV: The University of Texas Rio Grande Valley, UNITED STATES OF AMERICA

## Abstract

Environmental DNA (eDNA) offers valuable presence/absence data for populations and has been widely used in comprehensive biodiversity assessments. However, applying eDNA in terrestrial environments poses unique challenges, particularly in obtaining samples that are representative of ecological communities. eDNA extracted from top-predator dietary samples can be an effective sampling source in monitoring prey populations. In this study, we tested a novel, non-destructive protocol to assess the efficacy of eDNA from barn owl (*Tyto javanica delicatula*) pellets as a tool for monitoring small mammal communities in an arid environment. We assessed the species composition and abundance of small mammals from owl pellets collected in the Simpson Desert in far western Queensland, Australia, using a three-tiered approach. We extracted DNA from 50 owl pellets and targeted a 16S mini-barcode for metabarcoding. We compared species detection via genetic analysis with that of morphological analysis, and finally with historical small mammal trapping data. The DNA extraction method presented here resulted in full preservation of prey bones and fur material for museum archival. eDNA detected four mammal species that were not detected via morphological pellet analysis, three of which are significant detections that had not been observed at this location before but were expected to occur based on likely distribution ranges. However, a key limitation of the eDNA approach demonstrated in this study, is that taxonomic identification was constrained by the completeness of reference databases, which can result in false negatives or ambiguous assignments. The results of the present study demonstrate that the specificity of an eDNA approach can offer advantages compared with morphological identification of mammalian remains from owl pellets, and that genetic owl pellet analysis may be particularly useful in full vertebrate diversity assessments that include reptiles, birds and amphibians that are unidentifiable from skeletal remains.

## Introduction

Molecular diet analysis is a specialised form of environmental DNA (eDNA) analysis that uses genetic material from prey remains in diet samples, such as faeces or owl pellets, to identify consumed species [1,2]. While eDNA broadly refers to DNA shed by organisms into the environment with no associated tissue, molecular diet analysis deals with bulk material containing partially intact biological remains like hair, bones, or tissue [3,4]. Both approaches employ similar techniques to identify species from complex mixtures of fragmented DNA and fall under the umbrella of eDNA-based biodiversity assessment methods.

Advances in modern genomic techniques have enhanced the applicability of molecular-based assessments by enabling the analysis of even very small DNA fragments from environmental samples, providing data on the presence or absence of a species, or offering comprehensive biodiversity information for larger-scale studies (e.g., [5–7]). Thus, eDNA has become an increasingly popular tool for enhancing conservation efforts [8–10]. In particular, there is value in applying molecular-based methods in studies where environmental or logistical challenges make traditional ecological survey methods impractical or ineffective [11–14].

Environmental DNA techniques have become widely used for species detection and monitoring across both aquatic and terrestrial systems [15,16]. In terrestrial environments, eDNA-based approaches have taken many forms, including sampling soil, air, snow, from blood-feeding insects and dietary remains (e.g., faeces, regurgitated pellets and stomach contents) [13,17–19]. Molecular analysis of dietary samples can be used to study predator-prey interactions across a range of taxa, including birds, bats, rodents, and large carnivores [20–22]. These methods offer non-invasive alternatives for species detection and can provide valuable insights not only into trophic networks, but also into fluctuations in prey populations over time [23,24].

Small mammal populations are considered important indicators of ecological disturbances and are declining globally [25]. Active monitoring of these populations is imperative to manage and conserve the fauna and the landscapes they inhabit [26]. However, the integration of molecular dietary techniques into standardised small mammal monitoring and conservation frameworks remains limited. Standard practice for the monitoring of small mammal populations relies on methods that require direct observation of the target group (i.e., live-trapping), which can be invasive, time-intensive, logistically-constrained and expensive [27]. There is increasing scope to expand the role of molecular dietary analysis from investigating trophic interactions to be used more frequently in regular, systematic monitoring of small mammal populations, particularly in environments where traditional survey methods are logistically difficult or limited in taxonomic resolution.

Numerous studies have demonstrated the utility and applicability of morphological owl pellet analysis (i.e., prey species identification from undigested skeletal fragments) in various conservation and management scenarios, including monitoring and management of potential invasive species distributions [28], cryptic species detections [29] and whole mammalian diversity assessments [30–32]. However, there is potential for individual prey items to be misidentified or overlooked in owl pellets because body parts are crushed and disarticulated. The application of genetic techniques would not only strengthen the identification of mammal species’ presence, but also allow for the detection of potentially cryptic species and taxonomically challenging groups with subtle morphological differences.

Prey remains from owl pellets are subjected to high chemical degradation and DNA fragmentation during extensive owl digestion (similar to, although less degradation than faeces). As owls swallow their prey whole, digestible matter is stripped from prey bones via physical, enzymatic and acidic digestion [33,34]. Moreover, the pellets may have been deposited in areas exposed to a variety of environmental extremes and may be up to several years old. A successful genomic analysis protocol should allow for the detection of very degraded DNA fragments while retaining a high probability of identification to species level for the best study outcomes [35]. While genetic material from owl pellets has been used previously, the results of such studies are usually focused on taxonomic information regarding the owl [36,37] or single species prey detection using individual bone fragments [38,39]. Genetic technologies have only rarely been applied to the assessment of mammalian diversity [40–42] and never without the destruction of valuable remains required for archiving in museum reference collections [39,43,44].

We recently demonstrated the value of comparing traditional live-trapping data with morphological identification of species from owl pellets, highlighting the complementary strengths of these methods [28]. Building on these findings, the present study explores whether genetic analysis of owl pellet material can further improve species detection, potentially offering a more sensitive and comprehensive approach to biodiversity assessment in such challenging landscapes. Therefore, the present study aimed to develop and apply a non-destructive eDNA protocol for the analysis of remains from eastern barn owl (*Tyto javanica delicatula*) pellet material to monitor small mammal assemblages in far west Queensland, Australia. Specifically, we aimed to: (1) develop a non-destructive, whole owl pellet DNA extraction protocol; and (2) compare the ecological findings of the eDNA protocol versus morphological owl pellet ID and traditional live-trapping designs to determine the relative utility of the genetic method.

## Materials and methods

### Owl pellet sampling and live-trapping

Trapping and land access for this study was undertaken under Queensland Scientific Purposes Permit WISP18503317 (Queensland Government Department of Environment and Heritage Protection) and Animal Ethics approval CA 2019/07/1304 (Queensland Government Department of Agriculture and Fisheries Animal Ethics Committee).

We collected 185 owl pellets [28] from Pilungah Reserve, a Bush Heritage Australia owned and managed conservation reserve in far west Queensland, Australia (23.322766 S, 138.590459 E), to compare mammal species identification from morphological pellet analysis with historical live-trapping data (metal box and pitfall traps). Owl pellets were collected from the floor of a small cave in a rocky outcrop under the roost entrance and estimated to be up to five years old [28,45]. All pellets were collected in a single visit, stored together and dried before processing. A subset of five years of historical long-term live-trapping data from Pilungah Reserve [46] was used to compare results of genetic analysis with traditional methods (as per [28]).

For the present study, a random subset of 50 pellets from this collection was selected for genetic analysis as a proof of concept to evaluate the effectiveness of DNA-based species detection. Species detected through DNA analysis were compared to morphological identifications from the same pellets, as well as to five years of historical live-trapping data from the study area [28]. Additionally, results were cross-referenced with expected species based on known distributions, habitat preferences [47] and expert elicitation.

### Pellet processing and DNA extraction

Pellets were teased apart one-by-one, and identifiable skeletal material was removed for preservation and museum archiving. Morphological techniques were employed to identify individuals to species level where possible (see [28] for detailed methods).

DNA extraction was performed only on the remaining unidentifiable material (comprising of mostly fur and small bone fragments) using an adapted protocol based on the QIAGEN DNeasy Mericon Food Kit [48,49]. Unidentifiable remains of individual pellets were lysed in 50 mL falcon tubes overnight in 25 mL lysis buffer and 40 µL proteinase K at 60°C for a minimum of 10 hours [43,50]. Falcon tubes were spun down using a Beckman Coulter Allegra X-15R benchtop centrifuge at 4,500 g for 10 minutes to separate solid remains from the lysis buffer. 1 mL of lysis buffer was aliquoted from each sample tube and transferred to a fresh 2 mL tube then spun down again at 10,000 g for 5 minutes to separate any remaining fur or sediment. 700 µL of supernatant was transferred to a fresh 2 mL tube containing 500 µL chloroform as per the DNeasy Mericon Food Kit small fragment protocol. Steps 4 through to 12 of the protocol were followed according to manufacturer’s instructions. DNA yield of purified samples was quantified using a NanoDrop spectrophotometer.

### Sample pooling and sequencing

Purified samples were pooled in groups of five to lower the cost of subsequent sequencing. As all pellets were collected from the same site, at the same time, and the goal was to determine the list of species found in each sample pool, cross contamination between samples was not a concern. 5 μL of each pooled sample was aliquoted into a new sample tube (25 μL per sample) to generate 10 pooled samples (5 x 10 = 50 pellets total) for sequencing. Pooled samples were purified, concentrated and resuspended in 100 μL DNAse and RNAse free water using the Zymo Research DNA Clean & ConcentratorTM-25 kit to remove PCR inhibitors [24,51,52].

PCR was performed using KAPA HiFi Hotstart ReadyMix on final, pooled DNA extraction samples, targeting a mini barcode (148 bp) of the 16S region using a mammal-specific primer set (16Smam1, 16Smam2; [53]), synthesised with a 5’ addition of Illumina adapters (Table 1). These primers have been shown to be successful in highly degraded and ancient DNA samples [54], and thus, we deemed them appropriate to test against digested content from owl pellets. PCR conditions were optimised in a gradient PCR and thermal cycling was performed on pooled DNA samples following manufacturer’s instructions at 25 µL volumes with the following cycling conditions: 3 min at 95°C, followed by 30 cycles of 30 s at 95°C, 30 s at 65°C, 30 s at 72°C, with a final extension of 5 min at 72°C. PCR included positive and negative controls to ensure amplification reliability and integrity. PCR product was purified using JetSeqTM Clean magnetic beads at a bead to product ratio of 1:1 with two washes of fresh 80% ethanol following manufacturer’s protocol.

**Table 1:** 16Smam mammal primers (Taylor, 1996) and Illumina overhangs used to amplify mammal sequences from owl pellet remains.

	Primer Sequence	Illumina Overhang
16Smam1	5’-CGGTTGGGGTGACCTCGGA-3’	5’-TCGTCGGCAGCGTCAGATGTGTATAAGAGACAG-3’
16Smam2	5’-GCTGTTATCCCTAGGGTAACT-3’	5’-GTCTCGTGGGCTCGGAGATGTGTATAAGAGACAG-3’

Library preparation and sequencing were performed as per Illumina’s 16S protocol with Nextera indices. Samples were sequenced on a MiSeq V2 nano 300 cycle flow-cell to ensure >100,000 reads per sample.

### Anacapa pipeline adaptation

The Anacapa eDNA toolkit was adapted and used to assign Amplicon Sequence Variants (ASVs) to sequencing output for use in taxonomic assignment [55]. The Anacapa Toolkit was run in a singularity container on the Queensland University of Technology high performance computing system (HPC) with a maximum assignment of 64 GB memory, 24-hour wall-time and eight cores for each module.

### Customised CRUX reference database construction

The Anacapa Toolkit includes pre-built reference databases for eight commonly used metabarcoding loci. However, custom primers used in the present investigation required a customised database to suit. Therefore, before the standardised Anacapa workflow could be employed, a customised reference database was built using CRUX (Creating Reference libraries Using eXisting tools, [55]) to include all mammal species available on varying public databases (EMBL and NCBI). CRUX was first employed to create metabarcode-specific seed databases by querying the public databases [56]. Reference database sequences were filtered to contain only reads with robust taxonomic assignments (referring to tissue sample assignments only) [57]. Constructed databases were then converted for ecoPCR using the OBITools *obiconvert* command [58]. ecoPCR was used to run in silico PCR with the 16S mammal-specific primers used during wet lab PCR (excluding Illumina adaptors) to query the converted database and extract sequences to generate new, taxonomic reference libraries [59]. The *blastn* command from BLAST+ was then employed to query the new library against the NCBI RefSeq database [60] and retain only the longest version of each sequence using Entrez-qiime [61]. The locus-specific reference database was then ready for application in the Anacapa classifier module. To run CRUX, an adaptation was made to the config shell script file from [55] to include full paths to database input directories, and a new singularity image was written to include QIIME.

### Quality control

Raw sequence quality control was performed in the second Anacapa module. Primers and Illumina adapters were removed from raw Illumina fastq output files using cutadapt [62] and the FastX-toolkit [63]. Next, DADA2 [64] was employed to merge paired reads and generate ASVs.

The Anacapa classifier module was employed to query quality control-passed sequences against the CRUX database and assign taxonomy to ASVs. Bowtie2 [65] was used to BLAST individual ASV sequences against the CRUX reference database to extract the best 100 reference database hits (>95% identity and coverage) for each sample. To calculate confidence scores of database hits, a Bayesian Least Common Ancestor algorithm (BCLA; [66]) was then employed. First, a global multiple sequence alignment of the ASV sequences and its assigned Bowtie2 database hits was performed. 100 bootstrap samples were created from each hit sequence by replacement of single nucleotide bases. Posterior probability was calculated via a pairwise alignment of each hit and the original ASV sequence to determine the likelihood of observing the ASV sequence if it were derived from the taxa that each hit represents. The hit with the highest posterior probability was selected for each bootstrap sample (the “winner sequence”) and the number of times the winner sequence was selected out of all 100 bootstraps was counted to determine the confidence value of the taxonomic assignment (i.e., hit 1 = 80 counts, hit 2 = 20 counts: hit 1 is assigned with 80% confidence score). Thus, confidence scores represent the number of times out of 100 the taxonomic assignment was the best hit. This was repeated at each taxonomic level (species, genus, family, order, phylum, class and kingdom) to determine the confidence scores of taxonomic assignments across all levels. ASV tables were generated to include taxonomic assignment of each ASV with confidence scores at each taxonomic level.

To evaluate the validity of the Anacapa outputs, ASVs that returned expected species (based on known distributions) but with low confidence scores (<85% but >50%) were individually queried against the NCBI 16S reference database using a manual BLAST search [67] to corroborate taxonomic identity [68,69]. ASVs that returned taxonomic identities with low confidence scores (<50%) were considered low quality and excluded from further taxonomic assignment.

### Anacapa output analysis

ASV tables were analysed using R version 4.1.0 [70]. A searchable table of read counts of each ASV per sample was generated using the DT: DataTables package [71] that would act as a matrix for further analyses. Low abundance taxa (0.1% or n < 6 reads per sample) were removed from the ASV table to filter miss hits and false positives [13,72–74]. Next, known contaminant taxa were removed (i.e., *Homo sapiens* and *Bos* sp. likely contaminated during pellet transportation). Lastly, ASVs were filtered to a confidence level cut-off of 85% [75] to ensure only accurate taxonomic assignments were included in further analysis. Filtering was carried out using the ampvis2 R package [76]. Rarefaction curves (200 permutations) were generated using the *amp_rarecurve* function in ampvis2 for each genetic sample to determine species richness as a factor of sequencing depth (number of reads).

To compare genus richness detected per sample by DNA metabarcoding and traditional morphological analysis of owl pellets, we performed a paired Wilcoxon signed-rank test since the richness data per sample were not normally distributed (as determined by a Shapiro-Wilk test for normality). To ensure a robust and meaningful comparison, taxa were aggregated to the genus level, providing a consistent taxonomic unit across both methods. Taxonomic overlap (of genera) between the two methods was evaluated at the sample level using the Jaccard similarity index (defined by Intersection/ Union). The mean Jaccard similarity index was calculated across all samples. Additionally, species richness (S) was counted for each method to compare overall detectability at the species level.

## Results

The adapted lysis protocol resulted in complete physical recovery of all unidentifiable prey remains (small bones and fur) following DNA extraction. While no overt damage to the recovered bones was observed, formal assessment of potential microstructural impacts of the lysis conditions was not performed.

### Sequencing and filtering quality

**Fig 1:**
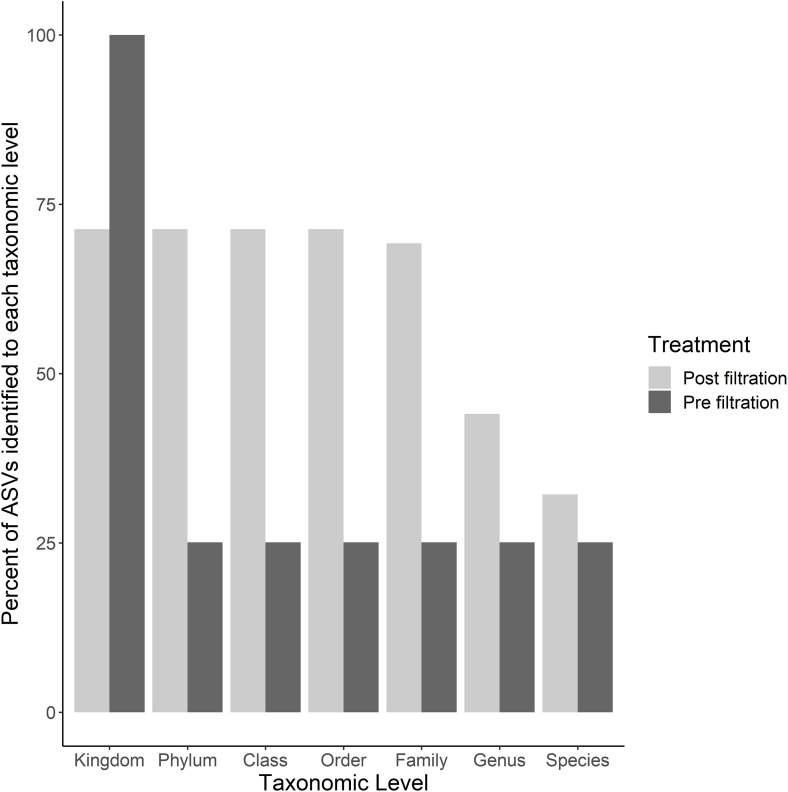
Percent of ASVs with taxonomic assignments across all taxonomic levels pre- and post-filtration by 85% confidence and minimum reads (n > 5).

**Fig 2:**
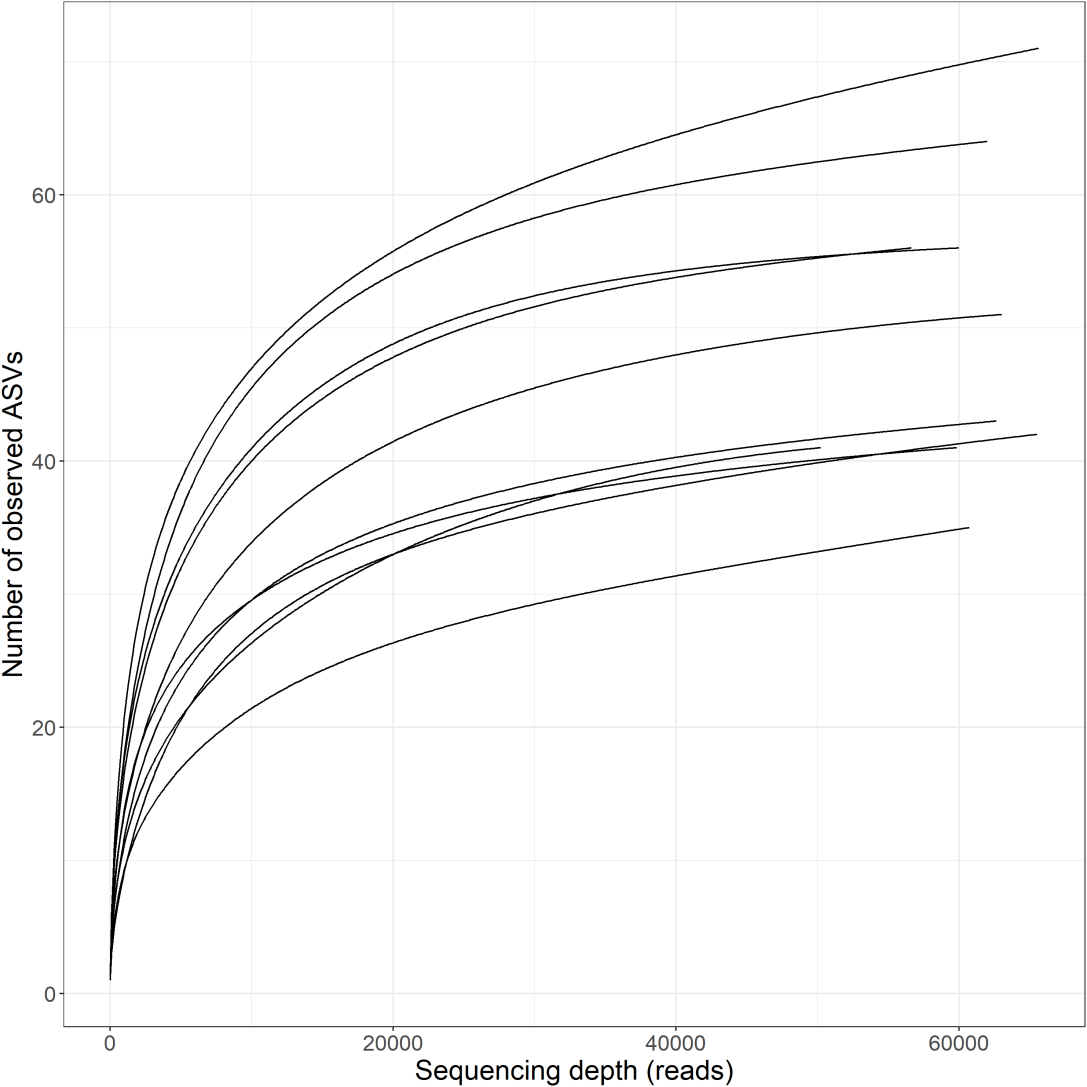
Rarefaction curves (100 permutations) depicting number of ASVs detected dependent on sequencing depth (no. of reads) after filtration.

MiSeq sequencing returned a total of 614,734 reads and 3,872 individual Amplicon Sequence Variants (ASVs) of which 25% were assigned taxonomy to species level before filtration (Fig 1). Filtration by 85% confidence and minimum read number (n > 5) removed ~97% of all ASVs and ~4,000 reads. A total of 137 ASVs remained across 610,241 reads in total across the 10 samples. The post-filtration data had a higher percentage of taxonomic assignments than the pre-filtration data across all taxonomic levels except that of kingdom (Fig 1). Sequencing depth was sufficient across all samples to reach maximum ASV richness (Fig 2, [76]).

### Ecological findings

**Fig 3:**
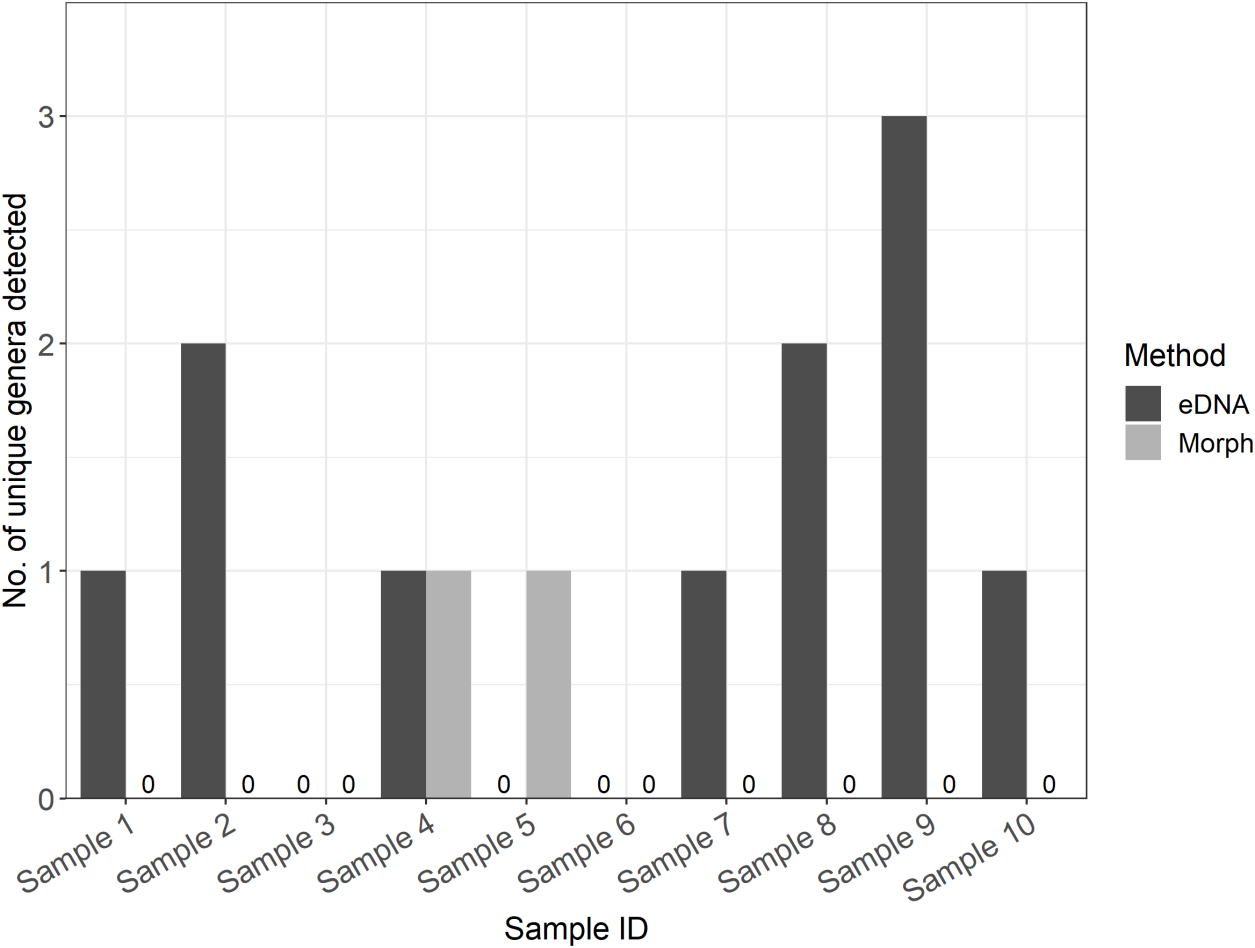
Number of unique small mammal genera detected per sample by both eDNA and morphological analysis. Complete overlap of one method with the other marked by ‘0’ values.

Genetic analysis detected a comparable number of genera per sample as traditional morphology (z = 2.5, p = 0.06), with a moderate to high taxonomic overlap (mean Jaccard similarity per sample = 0.69). However, genetic owl pellet analysis detected a higher number of unique genera per sample on average (i.e., genera that were detected by one method and not the other) than morphological analysis (n = 1.1, 0.2 respectively Fig 3).

Results returned a higher total species richness (S) for eDNA owl pellet analysis (S=8) compared to live-trapping (S=7) but lower than morphological owl pellet analysis (S=9) (Table 2). Five species identified through morphological analysis were not detected by genetic methods, likely due to incomplete reference databases. However, importantly, all species detected via morphological owl pellet analysis with representative 16S sequences on reference databases were also detected via genetic analysis with taxonomic assignments to species level (Table 2). Further, genetic analysis detected an extra three genera and four species that were not detected by morphological identifications, including: *Ningaui ridei*, *Pseudantechinus macdonnellensis*/*roryi*, *Rattus villosissimus* and *Zyzomys argurus*. *Pseudantechinus* sp. and *Rattus villosissimus* were detected via morphological owl pellet analysis in [28] indicating their presence at the site. These detections are of interest, as pellets containing these individuals were not included in the pooled samples used in the present subset data (S2 Table). Notably, *Pseudantechinus* sp. was not detected via morphological analysis in the pellets used in the present study (Table 2) but was detected in the overall batch of pellets collected from the site (see [28]). Nevertheless, *Pseudantechinus macdonnellensis* and *P. roryi* were returned with high probability after a manual BLAST search of our genetic data (Table 3).

**Table 2:** Detections of families, genera and species likely to occur at Pilungah Reserve based on known distribution [47] across the three survey methods. ^ indicates the taxonomic level is not represented in genetic reference databases. Shaded cells indicate species that were expected to occur at the chosen site but were not detected by any detection method.

Family	Genus/Genus species	Common name	eDNA	Morph.	Traps
**Dasyuridae**			**x**	**x**	**x**
	***Antechinomys***				
	*Antechinomys laniger*	Kultarr			
	***Dasycercus***		**x**	**x**	**x**
	*Dasycercus blythi*	Brush-tailed mulgara	x	x	x
	***Dasyuroides***				
	*Dasyuroides byrnei*	Kowari			
	***Ningaui***		**x**		**x**
	*Ningaui ridei*	Wongai ningaui	x		x
	***Planigale***				
	*Planigale tenuirostris*	Narrow-nosed planigale			
	***Pseudantechinus***		**x**		
	*Pseudantechinus macdonnellensis/roryi*	Fat-tailed false antechinus/Rory’s false antechinus	x		
	***Sminthopsis***		**x**	**x**	**x**
	*Sminthopsis crassicaudata*	Fat-tailed dunnart			
	*Sminthopsis hirtipes*	Hairy-footed dunnart			x
	*Sminthopsis macroura*	Stripe-faced dunnart	x	x	
	*Sminthopsis youngsoni*	Lesser hairy-footed dunnart	x	x	x
**Muridae**			x	x	x
	***Leggadina***		**x**		
	*Leggadina forresti ^*	Central short-tailed mouse			
	***Mus***		**x**	**x**	
	*Mus musculus*	House mouse	x	x	
	***Notomys*** *^*			**x**	**x**
	*Notomys alexis ^*	Spinifex hopping mouse		x	x
	*Notomys cervinus ^*	Fawn hopping mouse			
	*Notomys fuscus ^*	Dusky hopping mouse		x	
	***Pseudomys***		**x**	**x**	**x**
	*Pseudomys desertor ^*	Desert mouse		x	x
	*Pseudomys hermannsburgensis ^*	Sandy inland mouse		x	x
	***Rattus***		**x**		
	*Rattus villosissimus*	Long-haired rat	x		
	***Zyzomys***		**x**		
	*Zyzomys argurus*	Common rock rat	x		
**Vespertilionidae**			**x**	**x**	
	***Vespadelus*** *^*			**x**	
	*Vespadelus baverstocki ^*	Inland forest bat		x	
		**Families detected (of three total)**	**3**	**3**	**2**
		**Genera detected (of 14 total)**	**9**	**6**	**5**
		**Total species richness (S)**	**8**	**9**	**7**

**Table 3:** Manual NCBI database BLAST identities of ASV sequences with low confidence taxonomic assignment through Anacapa (<85%) with top-hit accession number.

ASV ID	Reads	Anacapa assignment	Confidence score	BLAST species	Identity	Accession no.
Merged_16Smam_8	893	*Sminthopsis macroura*	57.7%	*Sminthopsis macroura*	97.8%	JQ413951.1
Merged 16S_mam_59	6	*Rattus villosissimus*	76%	*Rattus villosissimus*	100%	GU570663.1
Forward_16Smam_26	7	*Pseudantechinus macdonnellensis*	62%	*Pseudantechinus macdonnellensis*	100%	MG251873.1
Reverse_16Smam_11	57	*Acomys cahirinus*	100%	*Zyzomys argurus*	100%	NC_057636.1
Merged_16Smam_35	42	*Neotoma isthmica*	100%	*Zyzomys argurus*	92.31%	NC_057636.1
Merged_16smam_41	22	*Praomys* sp.	100% (genus)	*Zyzomys argurus*	89%	NC_057636.1
Merged_16Smam_38	34	*Pseudantechinus roryi*	55%	*Pseudantechinus roryi*	95%	EU086653.1

For the genetic data, the level of taxonomic assignment varied depending on the representation of each taxon on the 16S reference databases. Eight species, nine genera and three families were detected via genetic owl pellet analysis, and Vespertilionidae (evening bats) was the only family detected with no genera represented in 16S databases (Table 2).

Individual manual BLAST searches of ASVs detected at high read frequency (n > 5) on NCBI databases returned an extra four taxonomic assignments to species level with an identity score greater than 85% (Table 3). Raw sequences for each ASV are attached in S1 Table. Manual BLAST searches corroborated (97.8–100% confidence) taxonomic assignments of two species (*Sminthopsis macroura* and *R. villosissimus*) that were initially returned with low Anacapa confidence scores (<85%). Interestingly, the dasyurid *P. macdonnellensis* was also detected via this secondary BLAST. A first pass through Anacapa modules detected the genus *Pseudantechinus* but without a confident taxonomic assignment to species level. However, manual BLAST searches querying the NCBI taxonomic database of two ASVs returned a 100% identity score to *P. macdonnellensis* and a second to *P. roryi* at 95% identity. Further, unlikely exotic rodent genera assigned by Anacapa (e.g., *Acomys* sp., *Neotoma* sp. and *Praomys* sp.) after manual BLAST all returned a high identity score (>85%) to the endemic common rock rat (*Z. argurus*), a species not detected by either morphological pellet analysis or live-trapping methods but predicted as a species that may occur at Pilungah [47].

eDNA diversity was dominated by the genera *Pseudomys* and *Mus*, which together accounted for the majority of total reads, while all other genera comprised only around 10% of total reads. When comparing taxonomic detections across the 10 samples using both morphological and eDNA methods, some discrepancies were observed. For example, *Dasycercus* was identified via morphological analysis in sample 5 (S2 Table) but was detected via eDNA in sample 10 (S3 Table). Even species represented by very few individuals in the morphological analysis (n < 5) were detected to species level through genetic analysis (e.g., *S. youngsoni* and *D. blythi*, both n = 1; Table 4). Additionally, although the genus *Vespadelus* was not represented in the reference database, eDNA analysis still detected the family Vespertilionidae, despite only one individual being identified morphologically.

**Table 4:** Number of individual specimens identified for each species through morphological analysis of pellet contents, alongside their detection via genetic analysis (eDNA). The Morphological analysis column shows counts of individuals physically identified, while the eDNA column indicates various detection confidence levels of genetic analysis due to incomplete reference sequences: x = species-level identification, * = genus-level identification, and ** = family-level identification.

Pooled samples contents	Morphological analysis	eDNA
*Pseudomys hermannsburgensis*	57	*
*Mus musculus*	54	x
*Sminthopsis macroura*	12	*
*Notomys* cf. *fuscus*	7	**
*Notomys alexis*	6	**
*Pseudomys desertor*	1	*
*Sminthopsis youngsoni*	1	x
*Dasycercus blythi*	1	x
*Vespadelus* cf. *baverstocki*	1	**

## Discussion

The present study has demonstrated that genetic owl pellet analysis is a valuable tool in detecting comparable prey taxa to morphological analysis, with species-level resolution constrained primarily by reference database coverage rather than by methodological sensitivity. Indeed, eDNA analysis allowed the identification of several species that went undetected via both live-trapping surveys and morphological analysis of owl pellets. However, the incompleteness of publicly available reference libraries does impose a limitation on species-level identification [3,77,78]. In the context of our study, genetic reference databases are lacking representation of 16S sequences for multiple mammalian taxa that might be expected at the study site. Most notable is the poor representation of some native Australian rodents. The genus *Notomys* (native hopping mice) is not represented at all, while the genus *Pseudomys* (native mice) is represented by only one species (*P. chapmani*) [79], with a distribution that does not coincide with the study area [47]. At the time of designing this workflow, 16S was considered the widely accepted barcode for Australian marsupials. While the marker worked well for broad taxonomic detection, the simultaneous construction of a custom reference database would have improved taxonomic resolution. Where resources and access to reference material permit, we recommend the use of various custom reference sequences alongside publicly available reference libraries (e.g., [80]). Furthermore, the use of multiple barcoding regions would also improve overall coverage of expected taxa (e.g., [5,7,81]). As more genetic studies are undertaken, public DNA databases will continue to improve and serve as an increasingly accurate reference database in metabarcoding studies.

The results of the present study also demonstrate the importance of evaluating pipeline-based genetic data outputs. Bioinformatic pipelines are an important aspect of eDNA analysis. Without them, the volume of data output by next generation sequencing data would be extremely difficult and time-consuming to analyse [82]. However, corroboration of pipeline outputs remains integral to the generation of accurate taxonomic assignments [68,83,84]. The present study utilised and adapted the Anacapa eDNA ToolKit [55] to query a customised mammalian reference database and assign taxonomy, but use of the pipeline alone failed to assign correct taxonomy to seven ASVs. When blasted manually against the NCBI reference database, however, these ASVs returned taxonomic assignments with high probability of correct identity (>85%). A possible cause for this discrepancy is the lack of reference sequences on public databases of the species of interest. For example, three ASVs that were assigned three different species, were resolved to *Z. argurus* with high identity via a manual BLAST search. *Zyzomys argurus* is only represented by one 16S sequence. Thus, the majority of database hits assigned in the BCLA algorithm were other species with similar genetic composition, which dominated the calculation of confidence scores and taxonomic assignments in the pipeline. Therefore, while eDNA pipelines are necessary for sequence quality control and data analysis of very large volumes of data, the current study demonstrates the importance of post-pipeline data evaluation, especially given the incomplete taxa representation and weighted bias prevalent in reference databases.

A notable finding of our study was that numerous species were detected to species level with high confidence via very small numbers of reads, demonstrating that the detection rate for assumed low abundance taxa in eDNA samples remains highly sensitive in taxonomic assignment and thus useful in presence/absence studies.

### Ecological implications

All taxa observed in owl pellets through morphological analysis were also detected via genetic analysis to either species or genus level (excluding species from unrepresented genera in the comparative database, *Vespadelus* and *Notomys*). Importantly, we detected an extra four mammal species that were not detected via morphological analysis of this batch of pellets. Of these four, two species had never been detected in live-trapping surveys, although their distributions suggested they may be present [47]. This demonstrates not only the value of owl pellet analysis in general, but of genetic analysis in particular, and the power of adopting a multi-faceted approach to ecological surveys when the focus is to attain accurate presence/absence data for mammal assemblages at a site.

The dasyurid *Pseudantechinus* cf. *mimulus* was observed in morphological owl pellet analysis of the whole batch of 185 pellets [28], but the pellets containing these individuals were not included in genetic analysis of the pooled subset of samples used in the current study. Due to its threatened status at the time, the observation of this species via morphological analysis in the total batch of pellets collected in 2018 was of considerable interest [28]. In the present study, the *Pseudantechinus* genus was detected as a direct output of the Anacapa pipeline, although species-level identification was not confirmed from direct outputs. However, further investigation, including a manual BLAST search of relevant ASVs, suggested the presence of *P. macdonnellensis* and *P. roryi* with high sequence identity to reference database representations. Based on the morphological identification of *P.* cf. *mimulus* [28], the eDNA-based detection of *P. macdonnellensis* and *P. roryi* were unexpected. The taxonomy of *P. macdonnellensis* and *P. roryi* remains unresolved, and this detection should therefore be further investigated using voucher specimens from the field site to examine more detailed genetics and morphology [79,85,86].

The successful detection of various small mammal taxa to species level in the present study is promising for future genetic-based monitoring of mammalian fauna. However, due to the limiting scope of the target region used in this study, any non-mammalian fauna were disregarded during analysis. The utility of this genetic data could be expanded through the application of more generic primer pairs. Indeed, minibarcode regions of the COI region could be used to detect a diverse range of taxa that includes mammals, reptiles and birds (e.g., [87]).

### Bias in owl pellet analysis and trapping surveys

Interestingly, the wongai ningaui (*N. ridei*) was detected via genetic owl pellet analysis although it was not observed morphologically among prey remains. We suggested that the absence of this dasyurid from pellets, that was found often in the parallel live-trapping surveys at the site, may be a result of ningaui microhabitat preferences that were not common within the owl’s search radius [28]. However, the detection of the species in genetic owl pellet analysis points to the presence of false negatives and potential misidentifications via morphological analysis of skeletal remains, or the degradation of these remains to a level at which they are no longer identifiable. In fact, skeletal remains from a single prey individual could be distributed across multiple pellets. In such cases, identifiable elements (e.g., mandibles) may be present in a different pellet than the one used for DNA extraction, while less diagnostic remains (fur, postcranial bone fragments, etc.) are present in the analysed sample. Therefore, a taxon detected via DNA but not morphology could be due to the absence of diagnostic (craniodental) skeletal elements in the specific pellet sampled.

Similarly, *Sminthopsis hirtipes* was observed only in live-trapping data and not in pellets, whereas *S. youngsoni* was detected in both morphological and genetic owl pellet analysis. These two dunnart species share many morphological characteristics and habitat preferences [47,88]; thus, the potential for misidentification in the field or laboratory is not trivial. Further, the native rock rat (*Z. argurus*) was detected via genetic owl pellet analysis and not via any other method. Importantly, this is the first time this species has been detected in the Simpson Desert. This is slightly outside of its known distribution but within its predicted area of occurrence, since the rocky habitat the species prefers is patchily present at Pilungah Reserve [47].

Genetic analysis is often relied upon for corroboration of taxonomic assignment through other means of identification [44,89,90]. This is especially true in cases where traditional identification systems are limited by time and/or ethical restraints (i.e., a live-capture in the field) [91,92], difficulty distinguishing characteristics between species of the same genus (high levels of expertise is sometimes required) [88] and missing key characteristics (i.e., missing teeth in skeletal remains) [93]. Our study demonstrates that using genetic techniques from the outset may help avoid the unnecessary double-handling of specimens that are challenging to identify through traditional morphological methods.

However, while owl pellet analysis offers valuable data on small mammal communities, it is important to acknowledge inherent potential biases related to the predator’s hunting behaviour and dietary preferences. Although barn owls are generally considered opportunistic hunters [94,95], some studies indicate that prey selection may favour certain size classes or may be dependent on hunting range habitat types [96,97]. For example, larger prey species may be underrepresented in pellets, while smaller species abundant in open habitats may be overrepresented, potentially skewing community composition assessments. Furthermore, dietary data reflect only species consumed by owls and thus exclude taxa not preyed upon, limiting the scope of biodiversity detected via this method. Sampling pellets from multiple individuals and roosts could mitigate some of these biases by capturing a broader prey spectrum [98].

Finally, while the specificity of eDNA offers clear advantages, it is important to interpret positive detections with caution. A positive DNA detection does not in itself constitute definitive evidence of species presence. Rather, metabarcoding should be viewed as a screening tool that is particularly valuable for identifying rare, cryptic, or hard-to-trap species that may otherwise go undetected. Where unexpected or significant taxa are identified, the original pellet can be revisited to search for corresponding skeletal remains. If found, the species’ presence can be confidently confirmed. If not, such detections can inform more targeted follow-up surveys, including habitat-specific trapping, additional pellet sampling, or species-specific qPCR assays. In this way, morphological and genetic analysis of owl pellets can serve as a valuable starting point for more focused ecological investigation and monitoring.

The present study is the first to demonstrate the efficacy of an adapted non-destructive, DNA extraction protocol from owl pellets for analysis of mammalian prey as a measure of mammalian richness. In previous work, we demonstrated the overarching validity and benefits of owl pellet analysis through morphological identification of skeletal prey remains when performed in conjunction with traditional ecological live-trapping surveys [28]. The present study built on this information and serves as an exploratory case study for the application of genetic techniques to further improve prey identification in terrestrial dietary analyses. Importantly, the present study demonstrates that the specificity of the eDNA approach offers advantages compared with the morphological identification of mammalian remains. But most of all, the detection of species through eDNA analysis that were not identified via morphological pellet analysis or live-trapping surveys highlights the growing value of eDNA as an essential tool in the field ecologist’s survey toolkit.

## Supporting information

S1 Table Raw sequences of ASVs BLAST manually on NCBI databases. (DOCX)

S2 Table Owl pellet contents as identified through morphological analysis of pooled DNA samples prior to eDNA analysis. Each pooled DNA sample comprised of five full owl pellet DNA extractions. Pellets highlighted in red signify that remains were not sufficient to identify to species level.
(DOCX)

S3 Table Relative abundance (%) of each genus and species detected via both morphological and genetic owl pellet analysis for each of the 10 pooled genetic samples. (DOCX)
