# Supplementary material for: Non-destructive environmental DNA extracted from owl pellet contents: A valuable tool for monitoring mammalian species richness
Source: PLoS One. 2026 Mar 9;21(3):e0344097. doi: 10.1371/journal.pone.0344097 (PMC12970871; doi:10.1371/journal.pone.0344097)
Supplement: S1 Table — (DOCX) [file pone.0344097.s001.docx]

**S1 Table. Raw sequences of ASVs BLAST manually on NCBI databases**

| **ASV ID** | **Sequence** |
| --- | --- |
| *Merged_16Smam_8* | GCATAAAACAACCTCCGAATGAAATAACCTAGATTTACCCATCTAAGTGTATTACTACCAGTAATTGACCCATTTTTTGATCAACGGAACA |
| *Forward_16Smam_26* | GCACAAAACAACCTCCGAATGAATATAACCTAGATTGACCAATCTAAGTGTAATACTACCAGTAATTGACCC |
| *Merged 16S_mam_59* | GAATAAAAAATCCTCCGAACGATTTTAACCTAGACCAACAAGTCAAAGTAATTCTAATATCCAATTGACCCAATTATTGATCAACGGACCA |
| *Merged 16S_mam_35* | ACAAAAAATCCTCCGCATGAATATAACCTAGACATACAAGTCAAAGTAAGTTATCTATCTTATTGACCCAAAAACTTGAGCAATGGACCA |
| *Merged 16S_mam_41* | GAACAAAAAAATCCTCCGAACGATTATAACAGAGACCTACAAGCCAAAGTAAGTTACCTATCTTACTGACCCAAAATATCTTGAACAATGGACCA |
| *Reverse_16Smam_11* | TGGTCCATTGATCAAGATTTTGGATCAATAAGATAGATAAC |
| *Merged_16Smam_38* | GCATAAAACAACCTCTGAATGAATAGAACCTAGATTAACCAATCTAAGTGTAACAATACCAGTAATTGACCCATCTCTTGATCAATGGAACA |
