# Supplementary material for: Non-destructive environmental DNA extracted from owl pellet contents: A valuable tool for monitoring mammalian species richness
Source: PLoS One. 2026 Mar 9;21(3):e0344097. doi: 10.1371/journal.pone.0344097 (PMC12970871; doi:10.1371/journal.pone.0344097)
Supplement: S2 Table — Each pooled DNA sample comprised of five full owl pellet DNA extractions. Pellets highlighted in red signify that remains were not sufficient to identify to species level. (DOCX) [file pone.0344097.s002.docx]

**S2 Table. Owl pellet contents as identified through morphological analysis of pooled DNA samples prior to eDNA analysis. Each pooled DNA sample comprised of five full owl pellet DNA extractions. Pellets highlighted in red signify that remains were not sufficient to identify to species level.**

| Sample ID | Pellet ID | DNA concentration (ng/μL) | ***Mus musculus*** | ***Pseudomys hermannsburgensis*** | ***Pseudomys desertor*** | ***Notomys alexis*** | ***Notomys cf. fuscus*** | ***Sminthopsis macroura*** | ***Sminthopsis youngsoni*** | ***Dasycercus blythi*** | ***Vespadelus cf. baverstocki*** |
| --- | --- | --- | --- | --- | --- | --- | --- | --- | --- | --- | --- |
| P1 | 7 | 69.1 | 2 | 1 |  |  |  | 1 |  |  |  |
|  | 4 | 56.2 | 2 | 1 |  | 1 |  |  |  |  |  |
|  | 72 | 50.6 |  | 1 |  |  |  |  |  |  |  |
|  | 60 | 49.8 |  |  |  |  |  |  |  |  |  |
|  | 5 | 46.1 | 1 | 3 |  |  |  | 3 |  |  |  |
| P2 | 91 | 45.8 |  | 2 |  |  | 1 |  |  |  |  |
|  | 14 | 45.6 |  | 1 |  | 1 |  |  |  |  |  |
|  | 105 | 41.3 |  | 2 |  |  |  |  | 1 |  |  |
|  | 6 | 40.6 |  | 1 |  |  |  | 1 |  |  |  |
|  | 1 | 37.9 | 3 | 2 |  |  |  | 1 |  |  |  |
| P3 | 13 | 32.9 | 3 |  |  |  |  |  |  |  |  |
|  | 16 | 32.6 |  | 1 |  |  |  |  |  |  |  |
|  | 61 | 32.3 | 3 |  |  |  |  |  |  |  |  |
|  | 11 | 30.2 | 2 |  |  |  |  |  |  |  |  |
|  | 12 | 28.4 |  |  |  |  |  |  |  |  |  |
| P4 | 40 | 27.8 | 1 | 3 |  |  |  | 3 |  |  |  |
|  | 62 | 27.7 | 2 |  |  |  |  |  |  |  |  |
|  | 34 | 23.3 |  | 1 |  |  |  |  |  |  |  |
|  | 42 | 23.1 |  | 1 |  |  |  |  |  |  |  |
|  | 10 | 20.2 | 1 | 1 |  |  |  |  |  |  |  |
| P5 | 38 | 18.7 | 1 |  |  |  |  |  |  |  |  |
|  | 37 | 18.6 | 3 | 1 |  |  |  |  |  |  | 1 |
|  | 100 | 17.3 |  | 1 |  |  |  |  |  | 1 |  |
|  | 104 | 13.8 | 3 |  |  |  |  | 1 |  |  |  |
|  | 86 | 12.8 | 2 | 3 |  |  | 1 |  |  |  |  |
| P6 | 117 | 11.5 |  |  |  |  |  | 1 |  |  |  |
|  | 27 | 11.2 | 2 | 3 |  |  |  |  |  |  |  |
|  | 30 | 11 |  | 2 |  |  |  |  |  |  |  |
|  | 24 | 8.2 | 2 | 3 |  |  | 1 |  |  |  |  |
|  | 28 | 8 |  | 2 |  |  |  |  |  |  |  |
| P7 | 124 | 7.8 | 1 | 1 |  |  |  |  |  |  |  |
|  | 64 | 7.2 | 1 | 2 |  |  |  |  |  |  |  |
|  | 53 | 6.9 |  | 2 |  | 2 |  |  |  |  |  |
|  | 36 | 6 | 2 | 1 | 1 |  |  |  |  |  |  |
|  | 20 | 5.6 | 1 |  |  |  |  | 1 |  |  |  |
| P8 | 58 | 5.4 | 2 | 3 |  |  |  |  |  |  |  |
|  | 111 | 5.3 | 1 | 2 |  |  |  |  |  |  |  |
|  | 41 | 5.1 | 3 |  |  |  |  |  |  |  |  |
|  | 112 | 5 | 2 | 1 |  |  |  |  |  |  |  |
|  | 110 | 4.9 |  |  |  |  |  |  |  |  |  |
| P9 | 70 | 4.7 |  | 1 |  |  | 1 |  |  |  |  |
|  | 31 | 4.6 |  | 1 |  |  |  |  |  |  |  |
|  | 51 | 3.9 | 3 | 1 |  |  |  |  |  |  |  |
|  | 52 | 3.9 |  | 4 |  | 1 |  |  |  |  |  |
|  | 2 | 3.7 | 1 | 1 |  |  | 1 |  |  |  |  |
| P10 | 21 | 3.7 |  |  |  |  |  |  |  |  |  |
|  | 88 | 3.7 |  |  |  |  | 1 |  |  |  |  |
|  | 102 | 3.6 | 3 |  |  |  | 1 |  |  |  |  |
|  | 3 | 3.5 | 1 | 1 |  | 1 |  |  |  |  |  |
|  | 96 | 3.2 |  |  |  |  |  |  |  |  |  |
