# Supplementary material for: Non-destructive environmental DNA extracted from owl pellet contents: A valuable tool for monitoring mammalian species richness
Source: PLoS One. 2026 Mar 9;21(3):e0344097. doi: 10.1371/journal.pone.0344097 (PMC12970871; doi:10.1371/journal.pone.0344097)
Supplement: S3 Table — (DOCX) [file pone.0344097.s003.docx]

**S3 Table. Relative abundance (%) of each genus and species detected via both morphological and genetic owl pellet analysis for each of the 10 pooled genetic samples.**

| **Sample ID** | **Species** | **Detection method** | **Relative abundance in each sample (%)** | |
| --- | --- | --- | --- | --- |
| Sample 1 | *Pseudomys chapmani* | eDNA | 95.777 |  |
| Sample 1 | *Pseudomys hermannsburgensis* | Morphological | 37.5 |  |
| Sample 1 | *Mus musculus* | Morphological | 31.25 |  |
| Sample 1 | *Sminthopsis macroura* | Morphological | 25 |  |
| Sample 1 | *Notomys alexis* | Morphological | 6.25 |  |
| Sample 1 | *Mus musculus* | eDNA | 4.196 |  |
| Sample 1 | *Leggadina lakedownensis* | eDNA | 0.02 |  |
| Sample 1 | *Zyzomys argurus* | eDNA | 0.005 |  |
| Sample 1 | *Pseudomys hermannsburgensis* | eDNA | 0 |  |
| Sample 1 | *Pseudomys desertor* | Morphological | 0 |  |
| Sample 1 | *Pseudomys desertor* | eDNA | 0 |  |
| Sample 1 | *Notomys alexis* | eDNA | 0 |  |
| Sample 1 | *Notomys fuscus* | Morphological | 0 |  |
| Sample 1 | *Notomys fuscus* | eDNA | 0 |  |
| Sample 1 | *Sminthopsis macroura* | eDNA | 0 |  |
| Sample 1 | *Sminthopsis youngsoni* | Morphological | 0 |  |
| Sample 1 | *Sminthopsis youngsoni* | eDNA | 0 |  |
| Sample 1 | *Dasycercus blythi* | Morphological | 0 |  |
| Sample 1 | *Dasycercus blythi* | eDNA | 0 |  |
| Sample 1 | *Vespadelus baverstocki* | Morphological | 0 |  |
| Sample 1 | *Vespadelus baverstocki* | eDNA | 0 |  |
| Sample 1 | *Leggadina lakedownensis* | Morphological | 0 |  |
| Sample 1 | *Ningaui ridei* | Morphological | 0 |  |
| Sample 1 | *Ningaui ridei* | eDNA | 0 |  |
| Sample 1 | *Pseudomys chapmani* | Morphological | 0 |  |
| Sample 1 | *Zyzomys argurus* | Morphological | 0 |  |
| Sample 1 | *Zyzomys argurus* | eDNA | 0 |  |
| Sample 2 | *Pseudomys chapmani* | eDNA | 86.813 |  |
| Sample 2 | *Pseudomys hermannsburgensis* | Morphological | 50 |  |
| Sample 2 | *Mus musculus* | Morphological | 18.75 |  |
| Sample 2 | *Sminthopsis macroura* | Morphological | 12.5 |  |
| Sample 2 | *Mus musculus* | eDNA | 12.468 |  |
| Sample 2 | *Notomys alexis* | Morphological | 6.25 |  |
| Sample 2 | *Notomys fuscus* | Morphological | 6.25 |  |
| Sample 2 | *Sminthopsis youngsoni* | Morphological | 6.25 |  |
| Sample 2 | *Ningaui ridei* | eDNA | 0.35 |  |
| Sample 2 | *Sminthopsis youngsoni* | eDNA | 0.258 |  |
| Sample 2 | *Zyzomys argurus* | eDNA | 0.065 |  |
| Sample 2 | *Leggadina lakedownensis* | eDNA | 0.042 |  |
| Sample 2 | *Zyzomys argurus* | eDNA | 0.003 |  |
| Sample 2 | *Pseudomys hermannsburgensis* | eDNA | 0 |  |
| Sample 2 | *Pseudomys desertor* | Morphological | 0 |  |
| Sample 2 | *Pseudomys desertor* | eDNA | 0 |  |
| Sample 2 | *Notomys alexis* | eDNA | 0 |  |
| Sample 2 | *Notomys fuscus* | eDNA | 0 |  |
| Sample 2 | *Sminthopsis macroura* | eDNA | 0 |  |
| Sample 2 | *Dasycercus blythi* | Morphological | 0 |  |
| Sample 2 | *Dasycercus blythi* | eDNA | 0 |  |
| Sample 2 | *Vespadelus baverstocki* | Morphological | 0 |  |
| Sample 2 | *Vespadelus baverstocki* | eDNA | 0 |  |
| Sample 2 | *Leggadina lakedownensis* | Morphological | 0 |  |
| Sample 2 | *Ningaui ridei* | Morphological | 0 |  |
| Sample 2 | *Pseudomys chapmani* | Morphological | 0 |  |
| Sample 2 | *Zyzomys argurus* | Morphological | 0 |  |
| Sample 3 | *Mus musculus* | Morphological | 88.888 |  |
| Sample 3 | *Mus musculus* | eDNA | 71.852 |  |
| Sample 3 | *Pseudomys chapmani* | eDNA | 28.144 |  |
| Sample 3 | *Pseudomys hermannsburgensis* | Morphological | 11.111 |  |
| Sample 3 | *Zyzomys argurus* | eDNA | 0.003 |  |
| Sample 3 | *Pseudomys hermannsburgensis* | eDNA | 0 |  |
| Sample 3 | *Pseudomys desertor* | Morphological | 0 |  |
| Sample 3 | *Pseudomys desertor* | eDNA | 0 |  |
| Sample 3 | *Notomys alexis* | Morphological | 0 |  |
| Sample 3 | *Notomys alexis* | eDNA | 0 |  |
| Sample 3 | *Notomys fuscus* | Morphological | 0 |  |
| Sample 3 | *Notomys fuscus* | eDNA | 0 |  |
| Sample 3 | *Sminthopsis macroura* | Morphological | 0 |  |
| Sample 3 | *Sminthopsis macroura* | eDNA | 0 |  |
| Sample 3 | *Sminthopsis youngsoni* | Morphological | 0 |  |
| Sample 3 | *Sminthopsis youngsoni* | eDNA | 0 |  |
| Sample 3 | *Dasycercus blythi* | Morphological | 0 |  |
| Sample 3 | *Dasycercus blythi* | eDNA | 0 |  |
| Sample 3 | *Vespadelus baverstocki* | Morphological | 0 |  |
| Sample 3 | *Vespadelus baverstocki* | eDNA | 0 |  |
| Sample 3 | *Leggadina lakedownensis* | Morphological | 0 |  |
| Sample 3 | *Leggadina lakedownensis* | eDNA | 0 |  |
| Sample 3 | *Ningaui ridei* | Morphological | 0 |  |
| Sample 3 | *Ningaui ridei* | eDNA | 0 |  |
| Sample 3 | *Pseudomys chapmani* | Morphological | 0 |  |
| Sample 3 | *Zyzomys argurus* | Morphological | 0 |  |
| Sample 3 | *Zyzomys argurus* | eDNA | 0 |  |
| Sample 4 | *Mus musculus* | eDNA | 92.296 |  |
| Sample 4 | *Pseudomys hermannsburgensis* | Morphological | 46.153 |  |
| Sample 4 | *Mus musculus* | Morphological | 30.769 |  |
| Sample 4 | *Sminthopsis macroura* | Morphological | 23.076 |  |
| Sample 4 | *Pseudomys chapmani* | eDNA | 7.68 |  |
| Sample 4 | *Zyzomys argurus* | eDNA | 0.0137 |  |
| Sample 4 | *Dasycercus blythi* | eDNA | 0.009 |  |
| Sample 4 | *Pseudomys hermannsburgensis* | eDNA | 0 |  |
| Sample 4 | *Pseudomys desertor* | Morphological | 0 |  |
| Sample 4 | *Pseudomys desertor* | eDNA | 0 |  |
| Sample 4 | *Notomys alexis* | Morphological | 0 |  |
| Sample 4 | *Notomys alexis* | eDNA | 0 |  |
| Sample 4 | *Notomys fuscus* | Morphological | 0 |  |
| Sample 4 | *Notomys fuscus* | eDNA | 0 |  |
| Sample 4 | *Sminthopsis macroura* | eDNA | 0 |  |
| Sample 4 | *Sminthopsis youngsoni* | Morphological | 0 |  |
| Sample 4 | *Sminthopsis youngsoni* | eDNA | 0 |  |
| Sample 4 | *Dasycercus blythi* | Morphological | 0 |  |
| Sample 4 | *Vespadelus baverstocki* | Morphological | 0 |  |
| Sample 4 | *Vespadelus baverstocki* | eDNA | 0 |  |
| Sample 4 | *Leggadina lakedownensis* | Morphological | 0 |  |
| Sample 4 | *Leggadina lakedownensis* | eDNA | 0 |  |
| Sample 4 | *Ningaui ridei* | Morphological | 0 |  |
| Sample 4 | *Ningaui ridei* | eDNA | 0 |  |
| Sample 4 | *Pseudomys chapmani* | Morphological | 0 |  |
| Sample 4 | *Zyzomys argurus* | Morphological | 0 |  |
| Sample 4 | *Zyzomys argurus* | eDNA | 0 |  |
| Sample 5 | *Mus musculus* | eDNA | 60.248 |  |
| Sample 5 | *Mus musculus* | Morphological | 50 |  |
| Sample 5 | *Pseudomys chapmani* | eDNA | 39.731 |  |
| Sample 5 | *Pseudomys hermannsburgensis* | Morphological | 27.778 |  |
| Sample 5 | *Notomys fuscus* | Morphological | 5.556 |  |
| Sample 5 | *Sminthopsis macroura* | Morphological | 5.556 |  |
| Sample 5 | *Dasycercus blythi* | Morphological | 5.556 |  |
| Sample 5 | *Vespadelus baverstocki* | Morphological | 5.556 |  |
| Sample 5 | *Zyzomys argurus* | eDNA | 0.021 |  |
| Sample 5 | *Pseudomys hermannsburgensis* | eDNA | 0 |  |
| Sample 5 | *Pseudomys desertor* | Morphological | 0 |  |
| Sample 5 | *Pseudomys desertor* | eDNA | 0 |  |
| Sample 5 | *Notomys alexis* | Morphological | 0 |  |
| Sample 5 | *Notomys alexis* | eDNA | 0 |  |
| Sample 5 | *Notomys fuscus* | eDNA | 0 |  |
| Sample 5 | *Sminthopsis macroura* | eDNA | 0 |  |
| Sample 5 | *Sminthopsis youngsoni* | Morphological | 0 |  |
| Sample 5 | *Sminthopsis youngsoni* | eDNA | 0 |  |
| Sample 5 | *Dasycercus blythi* | eDNA | 0 |  |
| Sample 5 | *Vespadelus baverstocki* | eDNA | 0 |  |
| Sample 5 | *Leggadina lakedownensis* | Morphological | 0 |  |
| Sample 5 | *Leggadina lakedownensis* | eDNA | 0 |  |
| Sample 5 | *Ningaui ridei* | Morphological | 0 |  |
| Sample 5 | *Ningaui ridei* | eDNA | 0 |  |
| Sample 5 | *Pseudomys chapmani* | Morphological | 0 |  |
| Sample 5 | *Zyzomys argurus* | Morphological | 0 |  |
| Sample 5 | *Zyzomys argurus* | eDNA | 0 |  |
| Sample 6 | *Pseudomys hermannsburgensis* | Morphological | 62.5 |  |
| Sample 6 | *Pseudomys chapmani* | eDNA | 60.248 |  |
| Sample 6 | *Mus musculus* | eDNA | 39.631 |  |
| Sample 6 | *Mus musculus* | Morphological | 25 |  |
| Sample 6 | *Notomys fuscus* | Morphological | 6.25 |  |
| Sample 6 | *Sminthopsis macroura* | Morphological | 6.25 |  |
| Sample 6 | *Sminthopsis youngsoni* | eDNA | 0.116 |  |
| Sample 6 | *Zyzomys argurus* | eDNA | 0.004 |  |
| Sample 6 | *Pseudomys hermannsburgensis* | eDNA | 0 |  |
| Sample 6 | *Pseudomys desertor* | Morphological | 0 |  |
| Sample 6 | *Pseudomys desertor* | eDNA | 0 |  |
| Sample 6 | *Notomys alexis* | Morphological | 0 |  |
| Sample 6 | *Notomys alexis* | eDNA | 0 |  |
| Sample 6 | *Notomys fuscus* | eDNA | 0 |  |
| Sample 6 | *Sminthopsis macroura* | eDNA | 0 |  |
| Sample 6 | *Sminthopsis youngsoni* | Morphological | 0 |  |
| Sample 6 | *Dasycercus blythi* | Morphological | 0 |  |
| Sample 6 | *Dasycercus blythi* | eDNA | 0 |  |
| Sample 6 | *Vespadelus baverstocki* | Morphological | 0 |  |
| Sample 6 | *Vespadelus baverstocki* | eDNA | 0 |  |
| Sample 6 | *Leggadina lakedownensis* | Morphological | 0 |  |
| Sample 6 | *Leggadina lakedownensis* | eDNA | 0 |  |
| Sample 6 | *Ningaui ridei* | Morphological | 0 |  |
| Sample 6 | *Ningaui ridei* | eDNA | 0 |  |
| Sample 6 | *Pseudomys chapmani* | Morphological | 0 |  |
| Sample 6 | *Zyzomys argurus* | Morphological | 0 |  |
| Sample 6 | *Zyzomys argurus* | eDNA | 0 |  |
| Sample 7 | *Pseudomys chapmani* | eDNA | 94.715 |  |
| Sample 7 | *Pseudomys hermannsburgensis* | Morphological | 40 |  |
| Sample 7 | *Mus musculus* | Morphological | 33.333 |  |
| Sample 7 | *Notomys alexis* | Morphological | 13.333 |  |
| Sample 7 | *Pseudomys desertor* | Morphological | 6.667 |  |
| Sample 7 | *Sminthopsis macroura* | Morphological | 6.667 |  |
| Sample 7 | *Mus musculus* | eDNA | 5.274 |  |
| Sample 7 | *Zyzomys argurus* | eDNA | 0.011 |  |
| Sample 7 | *Pseudomys hermannsburgensis* | eDNA | 0 |  |
| Sample 7 | *Pseudomys desertor* | eDNA | 0 |  |
| Sample 7 | *Notomys alexis* | eDNA | 0 |  |
| Sample 7 | *Notomys fuscus* | Morphological | 0 |  |
| Sample 7 | *Notomys fuscus* | eDNA | 0 |  |
| Sample 7 | *Sminthopsis macroura* | eDNA | 0 |  |
| Sample 7 | *Sminthopsis youngsoni* | Morphological | 0 |  |
| Sample 7 | *Sminthopsis youngsoni* | eDNA | 0 |  |
| Sample 7 | *Dasycercus blythi* | Morphological | 0 |  |
| Sample 7 | *Dasycercus blythi* | eDNA | 0 |  |
| Sample 7 | *Vespadelus baverstocki* | Morphological | 0 |  |
| Sample 7 | *Vespadelus baverstocki* | eDNA | 0 |  |
| Sample 7 | *Leggadina lakedownensis* | Morphological | 0 |  |
| Sample 7 | *Leggadina lakedownensis* | eDNA | 0 |  |
| Sample 7 | *Ningaui ridei* | Morphological | 0 |  |
| Sample 7 | *Ningaui ridei* | eDNA | 0 |  |
| Sample 7 | *Pseudomys chapmani* | Morphological | 0 |  |
| Sample 7 | *Zyzomys argurus* | Morphological | 0 |  |
| Sample 7 | *Zyzomys argurus* | eDNA | 0 |  |
| Sample 8 | *Pseudomys chapmani* | eDNA | 81.033 |  |
| Sample 8 | *Mus musculus* | Morphological | 53.333 |  |
| Sample 8 | *Pseudomys hermannsburgensis* | Morphological | 40 |  |
| Sample 8 | *Mus musculus* | eDNA | 18.751 |  |
| Sample 8 | *Sminthopsis macroura* | Morphological | 6.667 |  |
| Sample 8 | *Sminthopsis youngsoni* | eDNA | 0.187 |  |
| Sample 8 | *Zyzomys argurus* | eDNA | 0.029 |  |
| Sample 8 | *Pseudomys hermannsburgensis* | eDNA | 0 |  |
| Sample 8 | *Pseudomys desertor* | Morphological | 0 |  |
| Sample 8 | *Pseudomys desertor* | eDNA | 0 |  |
| Sample 8 | *Notomys alexis* | Morphological | 0 |  |
| Sample 8 | *Notomys alexis* | eDNA | 0 |  |
| Sample 8 | *Notomys fuscus* | Morphological | 0 |  |
| Sample 8 | *Notomys fuscus* | eDNA | 0 |  |
| Sample 8 | *Sminthopsis macroura* | eDNA | 0 |  |
| Sample 8 | *Sminthopsis youngsoni* | Morphological | 0 |  |
| Sample 8 | *Dasycercus blythi* | Morphological | 0 |  |
| Sample 8 | *Dasycercus blythi* | eDNA | 0 |  |
| Sample 8 | *Vespadelus baverstocki* | Morphological | 0 |  |
| Sample 8 | *Vespadelus baverstocki* | eDNA | 0 |  |
| Sample 8 | *Leggadina lakedownensis* | Morphological | 0 |  |
| Sample 8 | *Leggadina lakedownensis* | eDNA | 0 |  |
| Sample 8 | *Ningaui ridei* | Morphological | 0 |  |
| Sample 8 | *Ningaui ridei* | eDNA | 0 |  |
| Sample 8 | *Pseudomys chapmani* | Morphological | 0 |  |
| Sample 8 | *Zyzomys argurus* | Morphological | 0 |  |
| Sample 8 | *Zyzomys argurus* | eDNA | 0 |  |
| Sample 9 | *Pseudomys chapmani* | eDNA | 88.103 |  |
| Sample 9 | *Pseudomys hermannsburgensis* | Morphological | 53.333 |  |
| Sample 9 | *Mus musculus* | Morphological | 26.667 |  |
| Sample 9 | *Notomys fuscus* | Morphological | 13.333 |  |
| Sample 9 | *Mus musculus* | eDNA | 11.749 |  |
| Sample 9 | *Notomys alexis* | Morphological | 6.667 |  |
| Sample 9 | *Ningaui ridei* | eDNA | 0.116 |  |
| Sample 9 | *Leggadina lakedownensis* | eDNA | 0.028 |  |
| Sample 9 | *Zyzomys argurus* | eDNA | 0.003 |  |
| Sample 9 | *Pseudomys hermannsburgensis* | eDNA | 0 |  |
| Sample 9 | *Pseudomys desertor* | Morphological | 0 |  |
| Sample 9 | *Pseudomys desertor* | eDNA | 0 |  |
| Sample 9 | *Notomys alexis* | eDNA | 0 |  |
| Sample 9 | *Notomys fuscus* | eDNA | 0 |  |
| Sample 9 | *Sminthopsis macroura* | Morphological | 0 |  |
| Sample 9 | *Sminthopsis macroura* | eDNA | 0 |  |
| Sample 9 | *Sminthopsis youngsoni* | Morphological | 0 |  |
| Sample 9 | *Sminthopsis youngsoni* | eDNA | 0 |  |
| Sample 9 | *Dasycercus blythi* | Morphological | 0 |  |
| Sample 9 | *Dasycercus blythi* | eDNA | 0 |  |
| Sample 9 | *Vespadelus baverstocki* | Morphological | 0 |  |
| Sample 9 | *Vespadelus baverstocki* | eDNA | 0 |  |
| Sample 9 | *Leggadina lakedownensis* | Morphological | 0 |  |
| Sample 9 | *Ningaui ridei* | Morphological | 0 |  |
| Sample 9 | *Pseudomys chapmani* | Morphological | 0 |  |
| Sample 9 | *Zyzomys argurus* | Morphological | 0 |  |
| Sample 9 | *Zyzomys argurus* | eDNA | 0 |  |
| Sample 10 | *Mus musculus* | eDNA | 81.212 |  |
| Sample 10 | *Mus musculus* | Morphological | 50 |  |
| Sample 10 | *Notomys fuscus* | Morphological | 25 |  |
| Sample 10 | *Pseudomys chapmani* | eDNA | 18.266 |  |
| Sample 10 | *Pseudomys hermannsburgensis* | Morphological | 12.5 |  |
| Sample 10 | *Notomys alexis* | Morphological | 12.5 |  |
| Sample 10 | *Dasycercus blythi* | eDNA | 0.521 |  |
| Sample 10 | *Zyzomys argurus* | eDNA | 0.001 |  |
| Sample 10 | *Pseudomys hermannsburgensis* | eDNA | 0 |  |
| Sample 10 | *Pseudomys desertor* | Morphological | 0 |  |
| Sample 10 | *Pseudomys desertor* | eDNA | 0 |  |
| Sample 10 | *Notomys alexis* | eDNA | 0 |  |
| Sample 10 | *Notomys fuscus* | eDNA | 0 |  |
| Sample 10 | *Sminthopsis macroura* | Morphological | 0 |  |
| Sample 10 | *Sminthopsis macroura* | eDNA | 0 |  |
| Sample 10 | *Sminthopsis youngsoni* | Morphological | 0 |  |
| Sample 10 | *Sminthopsis youngsoni* | eDNA | 0 |  |
| Sample 10 | *Dasycercus blythi* | Morphological | 0 |  |
| Sample 10 | *Vespadelus baverstocki* | Morphological | 0 |  |
| Sample 10 | *Vespadelus baverstocki* | eDNA | 0 |  |
| Sample 10 | *Leggadina lakedownensis* | Morphological | 0 |  |
| Sample 10 | *Leggadina lakedownensis* | eDNA | 0 |  |
| Sample 10 | *Ningaui ridei* | Morphological | 0 |  |
| Sample 10 | *Ningaui ridei* | eDNA | 0 |  |
| Sample 10 | *Pseudomys chapmani* | Morphological | 0 |  |
| Sample 10 | *Zyzomys argurus* | Morphological | 0 |  |
| Sample 10 | *Zyzomys argurus* | eDNA | 0 |  |
